# Multiple Group Membership and Well-Being: Is There Always Strength in Numbers?

**DOI:** 10.3389/fpsyg.2017.01038

**Published:** 2017-06-21

**Authors:** Anders L. Sønderlund, Thomas A. Morton, Michelle K. Ryan

**Affiliations:** ^1^Social, Environmental and Organisational Research Group, Department of Psychology, University of ExeterExeter, United Kingdom; ^2^Department of Public Health, University of Southern DenmarkOdense, Denmark; ^3^Department of Human Resource Management and Organizational Behavior, University of GroningenGroningen, Netherlands

**Keywords:** social identity complexity, stigma visibility, identity compatibility, multiple identities, well-being

## Abstract

A growing body of research points to the value of multiple group memberships for individual well-being. However, much of this work considers group memberships very broadly and in terms of number alone. We conducted two correlational studies exploring how the relationship between multiple group membership and well-being is shaped by (a) the complexity of those groups within the overall self-concept (i.e., social identity complexity: SIC), and (b) the perceived value and visibility of individual group memberships to others (i.e., stigma). Study 1 (*N* = 112) found a positive relationship between multiple group membership and well-being, but only for individuals high in SIC. This effect was mediated by perceived identity expression and access to social support. Study 2 (*N* = 104) also found that multiple group memberships indirectly contributed to well-being via perceived identity expression and social support, as well as identity compatibility and perceived social inclusion. But, in this study the relationship between multiple group memberships and well-being outcomes was moderated by the perceived value and visibility of group memberships to others. Specifically, possessing multiple, devalued and visible group memberships compromised well-being relative to multiple valued group memberships, or devalued group memberships that were invisible. Together, these studies suggest that the benefits of multiple group membership depend on factors beyond their number. Specifically, the features of group memberships, individually and in combination, and the way in which these guide self-expression and social action, determine whether these are a benefit or burden for individual well-being.

## Introduction

Social and cultural diversity in most Western countries is expanding as a result of rising social mobility, immigration, and international trade and investment (UN, 2009). In response to this increasing diversity, the social contexts in which people operate are becoming more complex, and as a consequence individual self-concepts are also changing. Whereas individual self-definitions were historically embedded within stable local contexts (Karner, 2011), in modern globalized society – where individuals cross geographical, cultural, and social borders more than ever – people express themselves in terms of a broader and more shifting array of group memberships and social categories (Crisp and Hewstone, 2006). These include categories based on demographic groupings like gender, ethnicity, nationality, or profession as well as on specific opinions, preferences, and shared activities (Tajfel et al., 1979; Crisp and Hewstone, 2006). Importantly, these categories now intersect in novel and interesting ways. For example, the movement of people across traditional boundaries, such as women and ethnic minorities into professional domains from which they were previously excluded, has challenged traditional stereotypes and created new hybridized identities (Crisp et al., 2001). All of these changes have the potential to impact on how individuals conceptualize the self, which in turn is likely to affect individual well-being (Cross et al., 2003; Crisp and Hewstone, 2006). The focus of this paper is on the specific nature of this relationship.

A growing body of research suggests that multiplicity of the self is generally a good thing. For example, membership in multiple groups has been associated with not only improved emotional well-being (Binning et al., 2009; Jetten et al., 2010), but also mental and physical resilience (Jones and Jetten, 2011), quality of life and coping (Haslam et al., 2008), and stress and social adaptation (Iyer et al., 2009). These positive effects are commonly attributed to the idea that identifying with multiple social groups grounds people more firmly in their social world, and provides them with multiple connections to similar others (Haslam et al., 2008; Jetten et al., 2012). The meaning and social support that follow from these connections, in turn, provide resources from which individuals can draw personal strength, resilience, and guidance in terms of values, attitudes, and behavior (e.g., Jetten et al., 2015; Chang et al., 2016; Steffens et al., 2016). Because of these properties, the greater the number of group memberships one has access to, the better one is likely to function.

Despite demonstrations of the psychological value of multiple group memberships, the exact mechanisms through which these contribute to well-being remain unclear. Indeed, research in this area begs the question of whether the reported benefits accumulate as a direct, linear function of the number of groups to which a person belongs, or whether additional factors are also necessary for membership in multiple groups to lead to positive outcomes. Intuitively, the features of specific group memberships, and the varying meaning of these to the individual in his or her social context, should be at least as important for well-being as the sheer number of groups to which they belong. For example, other theoretical models propose that the uniqueness of the specific identities that make up an individual’s self-concept should moderate any relationship between multiple group membership and individual outcomes, including well-being (Roccas and Brewer, 2002). Within this framework, only membership in multiple, distinctive groups should increase the complexity of the self and through this increase the psychological resources available to the individual (see also Linville, 1987).

The psychological consequences of group membership are also likely to be contingent on the value attached to these – both by the individual and others around them. Illustrative of this point, belonging to a low-status or stigmatized group has been found to compromise a person’s well-being (Puhl and Brownell, 2006). While it might be that membership in additional groups could weaken the impact of stigma on the individual, this is likely to be true only if those alternative bases for self-definition are valued rather than equally stigmatized (e.g., Rydell and Boucher, 2009). Similarly, membership in groups that are perceived to be in conflict or otherwise incompatible with one another (e.g., identifying as a male college football player, say, and ballet dancer) may also negatively affect well-being (Brook et al., 2008; Miramontez et al., 2008) – that is, simultaneously striving to live up to the standards of groups that have opposing values is more likely to be a strain than a source of individual strength. While it may be the case that membership in additional groups could buffer against the impact of stigma on the individual, this is only likely to be true if those alternative bases for self-definition are valued rather than equally stigmatized (e.g., Rydell and Boucher, 2009).

In the ways described above, various lines of previous research suggest that any relationship between multiple group memberships and well-being is unlikely to be simple, positive, and independent of other factors. The core concern of this paper is to provide further empirical investigation of this point. Specifically, we argue – and show – that the features of identities within the individual self-concept (e.g., whether individual identities are distinctive or not; and whether multiple identities are compatible or incompatible), and within the individual’s social context (e.g., whether identities are socially valued or devalued; easily expressed or complicated) are important for shaping their psychological consequences when combined. Before presenting the two studies that test these ideas, we elaborate on the literature that we have already begun to sketch out above, as well as outline in more detail our own perspective.

### Social Identity Complexity

**FIGURE 1:**
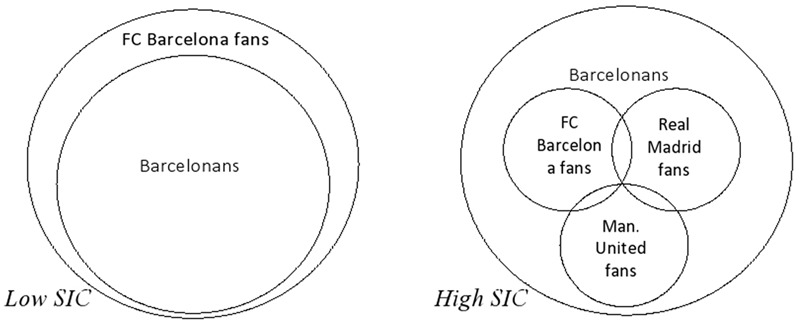
Graphic representation of social category membership overlap, indicative of low vs. high SIC.

As we have previously alluded to, the literature on (multiple) group memberships already contains theoretical frameworks that highlight the need to attend to the interrelationships among group memberships, rather than their sheer number alone. In particular, Roccas and Brewer (2002), have elaborated on the processes through which group membership might contribute to social identity complexity (SIC). From this perspective, SIC refers to the subjective perception of overlap between different self-defining groups or categories – that is, the degree to which different social groups or categories (and the identities associated with these) share members (Roccas and Brewer, 2002; Brewer, 2008; Schmid and Hewstone, 2011). This perceived overlap can depart significantly from more objective measures of category overlap (Schmid and Hewstone, 2011). As **Figure 1** illustrates, perceiving one’s social groups as highly overlapping may lead to the formation of a decidedly exclusive overall ingroup (for example, *all ‘real’ Barcelonans are FC Barcelona fans*), indicative of low SIC (Brewer and Pierce, 2005; Schmid and Hewstone, 2011). Alternately, others may perceive very little overlap, and recognize that ingroup members on one dimension are not necessarily ingroup members on another dimension (*some Barcelonans may be Real Madrid fans or Manchester United fans or neither or both*). This kind of representation would signify high SIC (Roccas and Brewer, 2002).

The SIC model was originally directed toward addressing questions about intergroup relations, rather than individual well-being. That is, high SIC was argued to be a source of positive intergroup perceptions and behavior because high SIC entails awareness of the cross-cutting relationships between groups. By contrast, the social cognition of those with low SIC is likely to be dominated by more simplified assumptions about group coherence and boundaries. Indeed, evidence suggests that high SIC is negatively associated with perceptions of intergroup threat, and prejudicial attitudes (e.g., Brewer and Pierce, 2005; Schmid et al., 2009). Although high SIC has also been suggested to be adaptive and beneficial in terms of individual well-being (Roccas and Brewer, 2002), to our knowledge, only limited research has been directed toward testing this possibility.

Nonetheless, SIC provides a useful theoretical starting point from which to explore the relationships between multiple group membership and well-being. As mentioned above, belonging to multiple distinct groups (high SIC) has been argued to be a source of identity strength, resilience, and well-being (Douglas, 2012; Jones et al., 2012). This may be because the more multifaceted and unique a person’s self-concept is, the richer and firmer their sense of self becomes. For example, an individual whose self-concept comprises many distinct, non-overlapping groups, likely has a broader and more varied foundation from which to access belonging and social support, and through this derive well-being (Jetten et al., 2015). Conversely, belonging to multiple overlapping groups, one does not have access to the spread of multiple distinct sources of identity, support, and connection, that might additively combine to improve overall well-being (Zimet et al., 1988; Jetten et al., 2012, 2015). Instead, this individual would have a relatively narrow and singular basis from which to conceptualize the self and on which to rely for group-based support and solidarity. In these ways, the SIC perspective elaborates on the factors that might shape how multiple group memberships relate to individual well-being. However, the exact question of how SIC translates into individual psychological outcomes (beyond intergroup orientations) remains relatively underdeveloped within this framework, and direct tests of this prediction are, at least to our knowledge, currently absent from the literature.

### Social Identity Compatibility and Stigma

Although the SIC framework provides a solid foundation for investigating the well-being consequences of multiple group membership, the complexity approach nonetheless maintains a focus on the number of individual group memberships (albeit in conjunction with their distinctiveness) as a primary determinant of individual psychological outcomes. The broader meaning of these group memberships – both to the individual and within their social context – is not explicitly theorized in this framework. Nonetheless, it seems plausible that factors associated with the meaning of identities – both in the eyes of the individual and of others – should play a role in determining links between multiple group memberships and well-being. For example, belonging to multiple, distinctive but devalued groups should be a different experience than belonging to multiple, distinctive, and valued groups – although both these configurations are high in identity complexity. To unpack this idea, we now turn to the literature on stigma and well-being.

Stigmatization has been found to have significant adverse impacts on psychological well-being in terms of depression (Lee et al., 2002; Lewis et al., 2003), reduced life satisfaction (Markowitz, 1998), anxiety and hopelessness (Lee et al., 2002), and psychological distress (Kang et al., 2006). While there are some studies demonstrating that the negative impact of stigma can sometimes be ameliorated – for example, when individuals can attribute their stigma to the prejudice of others rather than their own perceived deficits (Major and O’Brien, 2005) – the majority of research on the topic has demonstrated an adverse impact of stigma on psychological and physical health outcomes (Carr and Friedman, 2005; Ellemers and Barreto, 2006; Beals et al., 2009; Puhl and Heuer, 2009; Schmitt et al., 2014).

Notwithstanding the overall negative impact of stigmatized group memberships on well-being, some studies have suggested that multiplicity of the self could mitigate these negative effects (Mussweiler et al., 2000). To explain this pattern, it has been theorized that when any specific aspect of the self comes under threat or is devalued, individuals who have multiple selves on which to draw can more readily shift away from the threatened identity to an alternative one (Mussweiler et al., 2000; Benet-Martínez and Haritatos, 2005; Rydell et al., 2009; Sacharin et al., 2009; Jetten et al., 2012). Thus, multiple identities may *buffer* the individual against the effects of stigma attached to any single identity. On the other hand, an individual who belongs to a stigmatized group, but who has few alternative group memberships to draw on to define their self, is unlikely to be able to so easily disengage from the implications of their stigmatized group membership (Linville, 1987; Mussweiler et al., 2000; Roccas and Brewer, 2002).

Contrary to the idea of a buffer effect protecting the self-concept, however, research also suggests that belonging to a stigmatized group can preclude the individual from accessing the benefits associated with alternative group memberships. In particular, a stigmatized group membership might eclipse all other group memberships, and in this way dominate, rather than be cushioned by them. This is because stigmatized identities can become highly central to the self and to one’s interactions with others (Goffman, 1963). Specifically, due to their heightened salience, stigmatized identities can complicate the process of connecting to other groups and of maintaining meaningful relationships with others (Goffman, 1969; Goldstein, 2002). For example, being Black in a majority White (and perhaps racist) society can both restrict access to other social categories (e.g., occupation, community, education, etc.), and prevent the individual from being perceived accurately by others in terms of their other identities. Someone may be a doctor, a father, and a football fan, but the fact that he is also Black may ‘blind’ others to these additional group memberships, effectively undermining expression of these identities and thus inhibiting active participation in the associated social categories. This form of “identity constraint” might compromise the benefits of multiple group membership and place limits on individual well-being (Postmes and Branscombe, 2002). Indeed, past research has indicated that well-being is related to the extent to which the individual feels that he or she can self-express, and, in effect, be perceived by others accurately (Swann et al., 1989).

Stigmatized identities may also become a source of internal conflict and thereby place limits on the extent to which the individual can achieve a clear and cohesive self-concept. For instance, the group memberships of *woman* and *midwife* would be considered by most as highly compatible due to the fact that this profession is perceived as traditionally feminine and its workforce is predominantly female (Dimond, 2002). By contrast, being female and a doctor, or male and a midwife, may be considered more incompatible – and indeed the unexpected intersection of these categories can itself be a source of stigma (e.g., Purdie-Vaughns and Eibach, 2008; Meeussen et al., 2016; von Hippel et al., 2016; Veldman et al., 2017). Integrating these ideas with the multiple group membership literature, Brook et al. (2008) found that identity quantity (i.e., multiple group memberships) was positively correlated with well-being, but only when the specific identities involved were both important to the individual and perceived to be compatible with one another. When important identities were instead perceived to be incompatible, multiple identities were associated with reduced well-being. Thus, the compatibility of group memberships within the individual’s overall self-concept appears to structure the implications of these for individual well-being. In this way, we argue that both the perceived value of group memberships to others (i.e., stigma) and the sense of incompatibility that can arise from membership in multiple stigmatized groups, should modify previously observed links between multiple group memberships and well-being.

### The Present Research

While previous research clearly demonstrates a connection between multiple group membership and a range of well-being factors, we argue that this relationship is likely to be complex rather than straightforward, and contingent on a variety of additional factors. In the preceding sections, we identify two general areas of identity perception and integration that are likely to moderate the relationship between multiple group memberships and well-being in some way. These include the configuration of various identities within the self-concept (i.e., complexity), and the social value attached to those identities (i.e., stigma). These things may shape the degree to which possessing multiple group memberships supports (vs. undermines) individual action and interaction in the world, and the degree to which this allows for multiple group memberships to contribute to (vs. interfere with) the subjective cohesiveness of the individual’s self-concept. Further, being able to effectively articulate and express the self – both to others and within one’s own mind – and to access the support of others by virtue of their shared group membership, should all in turn contribute to enhanced individual well-being and thus mediate effects of multiple group memberships on the self.

To explore the relationship between multiple group membership and well-being in these terms, we conducted two correlational survey studies. The first study looked specifically at the impact of SIC on the relationship between multiple identities and well-being, while the second study investigated the significance of identity stigma and compatibility in this relationship. Across both studies, and consistent with previous literature, we expected that belonging to multiple groups would generally be associated with increased well-being. However, we also expected that this relationship would be amplified when those multiple group memberships were relatively distinctive (i.e., high SIC), and attenuated when group memberships were instead highly overlapping. This hypothesis was tested in Study 1.

Thinking about the relationship between multiple group membership and well-being in other terms than those connected to SIC, we expected that the perception of one’s identities as either devalued or valued within one’s social environment would moderate links between multiple group memberships and well-being. Specifically, the translation of (multiple) group memberships to positive well-being should be more straightforward when the individual identities are valued, or when devalued identities are not visible to others. In the context of multiple, visibly devalued (i.e., stigmatized) identities, relationships between multiple group membership and well-being would be negative. This hypothesis was tested in Study 2. Further, we also tested whether identity compatibility moderated the relationship between multiple group membership and well-being. That is, in line with past research (Brook et al., 2008), we believed that multiple identities would facilitate individual well-being, but only when those identities were compatible. This hypothesis was also tested in Study 2.

Finally, we explored plausible mediators of multiple group membership effects on well-being. In line with the above discussion, we explored perceived identity expression (i.e., the successful articulation of the self to others) and social support as potential mediators in both studies. At the outset of Study 2, and to be as comprehensive as possible, we supplemented the measure of social support to also include perceptions of social inclusion by others.

## Study 1

We conducted a correlational survey study designed to examine the relationship between multiple group membership and well-being. In addition to assessing the number of groups to which individuals belonged, we also assessed the perceived overlap vs. distinctiveness of the most important groups. Consistent with models of SIC (Roccas and Brewer, 2002), we hypothesized that multiple group membership would be positively associated with well-being when the key component identities are seen to be relatively distinct (high SIC) as opposed to overlapping (low SIC).

We were also interested in the mechanisms through which these effects might occur. Specifically, we reasoned that membership in multiple groups might, in fact, lay the foundations for a more distinctive sense of self and more practice enacting and expressing this self to others within one’s social environment. That is, if the individual perceives his or her multiple social identities as discrete and separate (high SIC), then this should translate into a clearer idea of exactly what these identities represent (both individually and in combination), ultimately facilitating their accurate and effective expression. By contrast, if the individual’s social identities are highly overlapping and indistinct (low SIC), communicating their precise content and meaning effectively to others may be relatively difficult. Accordingly, we measured perceived identity expression (the perceived ease and freedom to express and enact one’s identities to others). Finally, other research has highlighted the role of multiple group membership in providing individuals with access to multiple actual, or expected, bases of social support and thus well-being (Haslam et al., 2008). Here, we believe that belonging to multiple distinct groups (high SIC) translates into a corresponding number of distinct channels through which unique group-based social support may be accessed. Belonging to multiple overlapping groups, however, would likely offer relatively few distinct points of social support, ultimately comprising a narrow and redundant social support base. On this rationale, we also included a measure of social support as a mediator between SIC and individual well-being.

### Method

#### Participants

The research was conducted via an online survey advertised with flyers at various public locations (e.g., public transport, libraries, and universities), on social networking sites (linkedIn, facebook), as well as by email to personal and professional contacts. In response to this advertising, a sample of 131 adults was recruited. Of these, 19 cases had missing data and were therefore excluded from the analyses. The final sample of 112 participants included 23 males and 89 females. The majority of these (31.9%, *n* = 36) were aged between 18 and 25 years old, with a total of 68.2% (*n* = 77) of the sample being 18–35 years old. A total of 19 different nationalities were included in the sample, with the majority 75.3% (*n* = 85) being from Western countries such as Australia (31.9%, *n* = 36), the UK (30.1%, *n* = 34), or the US (13.3%, *n* = 15). The most common occupation was university student (58.6%, *n* = 65) followed by academic (15.3%, *n* = 17). The sample included 13 different ethnicities, but the vast majority of participants identified as White (75.9%, *n* = 85).

#### Survey and Measures

The survey comprised measures relating to identity and well-being.^1^ Specifically, the survey first asked participants to list as many group memberships that they could think of (‘In the text box below, list as many groups that you can think of that are relevant to your daily life’). From the resulting list, they were then prompted to choose the four groups that they felt were the most important and that best defined them. We decided to ask participants to choose four group memberships (as opposed to more or less), mainly for practical reasons to do with survey length, but also because four groups allow for multiple (specifically six) comparisons between individual group memberships (see Brewer and Pierce, 2005, for a similar method). Participants then responded to a series of items focusing on various aspects of social identity (complexity, expression, and centrality) in relation to each of the four groups they had chosen (detailed below). Participants finally completed more general measures of psychological well-being.

##### Identity measures

*Social identity complexity* was defined in terms of both the number of group memberships and the perceived extent of overlap between their most important (i.e., top four) groups (Roccas and Brewer (2002). A larger number of non-overlapping identities indicated higher identity complexity, whereas fewer and more overlapping identities indicated lower complexity. Thus, after listing any number of identities which defined them, each participant was asked to rate the degree of overlap between each possible pairing of his or her four most important identities (i.e., ‘of people who belong to e.g., the group American, how many also belong to the group Christian?’) on a 10-point Likert scale (1 = very few, 10 = nearly all). The average overlap score for all identity pairings was then calculated to obtain an overall measure of identity overlap.

We also measured a number of features of each of these identities (all measured on 5-point Likert scales with 1 = strongly disagree, 5 = strongly agree). First, *identity centrality* was measured with two items created for the study (‘The group [X] is an important reflection of who I am,’ ‘In general, belonging to [Group X] is an important part of my self-image’; α = 0.84). Next, *identity value* was rated both in terms of value to the self (‘To what extent do you consider your membership with [Group X] as generally positive or negative?’) and perceived value in the eyes of others (‘To what extent do you think your membership with [Group X] is considered positively or negatively by others in the community/society in which you live?’). These measures were included to give an indication of the nature of the identities selected by participants.

Finally, after rating each of their chosen four identities on each of these dimensions, a number of more general questions about the self and identity were asked that did not refer to the specific groups. These items were again measured on 5-point Likert scales ranging from 1 (strongly disagree) to 5 (strongly agree). First, three items were developed for this study to measure *perceived identity expression*. These items focused on the person’s perceived freedom to express their own identities, and the degree to which others accurately recognized how they saw themselves as a result (‘In general, I feel free to fully express myself and my identity to the people around me,’ ‘Other people don’t see me the way I want to be seen’ (reversed), and ‘Sometimes I feel like other people are trying to put me in a box that doesn’t fit’ (reversed; scale reliability α = 0.69).

##### Well-being measures

Psychological well-being was assessed through the General Well-being Index (GWBI) (Hopton et al., 1995) (scale reliability α = 0.89) using five multiple choice answer options (e.g., Q: ‘In general, do you feel disheartened or sad?’ A: ‘All of the time,’ ‘Most of the time,’ ‘From time to time,’ ‘Very occasionally,’ ‘Not at all’). A three-item measure of perceived access to *social support* was developed for the study and measured on 5-point Likert scales (scale reliability α = 0.82), ‘To what extent do you feel that you have family or friends so close to you that you can count on them if you have serious problems?’, ‘How much concern/interest do people show in what you are doing?’, ‘How difficult would it be for you to get practical help from neighbors if you should need it?’.

### Results

#### Descriptive Findings

On average, respondents listed a total of approximately seven (*M* = 7.05, *SD* = 3.18) groups that they believed defined them in some way. The nature of the groups reported varied widely among participants and included gender, sexuality, hobby, profession, ethnicity, etc. The perceived overlap between participants’ most important group memberships was generally low (*M* = 3.93, *SD* = 1.95 on a 10-point scale), indicating relatively high SIC. The identity and well-being variables yielded average scores significantly higher than the scale midpoint of 3.00 (see **Table 1**). Thus, participants were generally thinking about important, positively valued group memberships.

**Table 1:** Correlations between identity and well-being measures.

Variable	Mean	*SD*	1	2	3	4	5	6	7	8
(1) Identity quantity	7.05	3.18	-	0.15	0.12	-0.15	-0.05	0.02	0.17	0.15
(2) Identity overlap	3.93^†^	1.95		-	0.13	-0.12	-0.21^∗^	0.13	0.10	0.05
(3) Identity centrality	3.96^†^	0.66			-	0.22^∗^	0.07	0.09	-0.07	0.15
(4) Identity value (self)	4.26^†^	0.61				–	0.57^∗∗^	0.00	0.25^∗∗^	0.29^∗∗^
(5) Identity value (others)	3.90^†^	0.69					–	0.12	0.29^∗∗^	0.23^∗^
(6) Id. expression	3.37^†^	0.96						–	0.43^∗∗^	0.37^∗∗^
(7) Social support	3.96^†^	0.85							–	0.39^∗∗^
(8) Well-being	3.69^†^	0.85								–

*^∗^*p* < 0.05, ^∗∗^*p* < 0.01. ^†^Mean departs from scale midpoint (identity overlap = 5.5; all others = 3) significantly at *p <* 0.001. Identity overlap was measured on a 10-point Likert scale; all other measures were taken on 5-point scales.*

Next, mean correlations for the main identity and well-being variables were calculated to provide a preliminary assessment of any statistically significant relationships (see **Table 1**). Aside from a significant correlation between identity overlap and identity value (others), indicating that people with more overlap between identities (low complexity) perceived their group memberships as less socially valued, there were no significant correlations between identity overlap and any of the well-being measures. Nor were there any significant correlations between identity quantity and any other variable (see **Table 1**). To account for any potential variation within each mean identity measure, we also examined the association between the identity and well-being variables at the level of each individual identity that participants listed (i.e., their four most important identities). Here, we found no significant correlation between any measure of identity overlap, identity centrality, or identity visibility, and any of the outcome variables. Identity 2 and 4, however, did correlate significantly, but moderately, with social support, social inclusion, and well-being (see **Table 2**).

**Table 2:** Correlations between identity and well-being measures.

Variable	Level	Social support	Social inclusion	Perceived identity expression	Well-being
Identity overlap	Identity 1, 2	0.07	0.00	0.08	0.10
	Identity 1, 3	-0.08	-0.06	0.10	-0.01
	Identity 1, 4	0.04	0.19	0.18	-0.05
	Identity 2, 3	0.19	0.03	0.08	0.13
	Identity 2, 4	0.12	0.11	0.02	-0.03
	Identity 3, 4	0.09	0.00	0.02	0.04

Identity centrality	Identity 1	-0.10	0.01	0.09	0.11
	Identity 2	-0.05	-0.06	0.02	0.10
	Identity 3	-0.06	-0.06	0.04	0.07
	Identity 4	-0.13	-0.14	0.05	0.01

Identity visibility	Identity 1	0.03	0.07	0.03	0.03
	Identity 2	-0.10	-0.17	0.10	0.01
	Identity 3	0.02	-0.10	0.05	-0.01
	Identity 4	0.07	-0.02	0.02	-0.18

Identity value	Identity 1	0.15	0.18	-0.02	0.14
	Identity 2	0.19^∗^	0.22^∗^	0.08	0.29^∗^
	Identity 3	0.06	0.06	-0.05	0.12
	Identity 4	0.25^∗^	0.27^∗^	0.04	0.24^∗^

*^∗^Correlation is significant at the 0.05 level (two-tailed).*

#### Regression Analyses

Regression analyses were conducted to further ascertain the nature of the relationship between the variables, and to test the specific hypothesis that identity complexity (the combination of multiple, distinctive groups) has consequences for individual well-being. To test this hypothesis, well-being was used as the main dependent variable (DV), while identity overlap, identity quantity, and their interaction were entered as the independent variables (IV). Prior to the analysis, identity overlap and identity quantity were mean centered and an Overlap X Quantity interaction variable was computed by multiplying the centered scores. Regression analyses were conducted in which the main effect terms were entered at the first step, followed by the interaction term at Step 2.

##### Well-being

**FIGURE 2:**
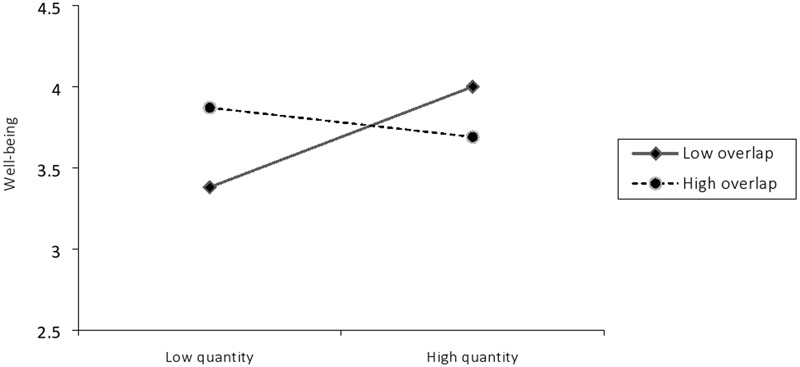
Interaction between identity quantity and overlap on well-being.

A regression analysis assessing the impact of identity overlap and identity quantity on well-being revealed no significant main effects (*β* = 0.06, *p* = 0.57, 95% CI low = -0.05, high = 0.11; *β* = 0.11, *p* = 0.25, 95% CI low = -0.03, high = 0.07, respectively). Inclusion of the interaction term, however, increased the overall variance explained, *R^2^* ^change^ = 0.05, *p* = 0.02, and the interaction itself was significant at this step, *β* = -0.22, *p* = 0.02, 95% CI low = -0.05, high = -0.00. In order to deconstruct the interaction, the effect of identity quantity was examined at high (+1 SD) and low (-1 SD) identity overlap (see **Figure 2**). This revealed a significant main effect for identity quantity at low identity overlap, *β* = 0.31, *p* = 0.02, 95% CI low = 0.05, high = 0.39, while no such effect was evident at high identity overlap, *β* = -0.09, *p* = 0.47, 95% CI low = -0.14, high = 0.09. Thus, it would appear that multiple group memberships are associated with enhanced well-being only at low levels of identity overlap (i.e., high complexity).

##### Perceived identity expression

**FIGURE 3:**
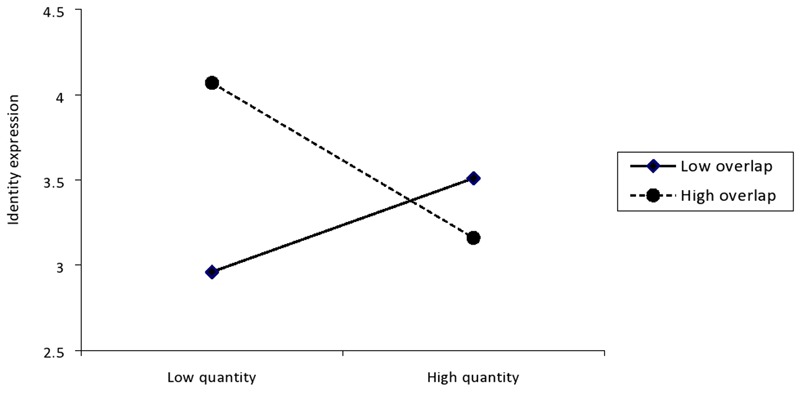
Interaction between identity quantity and overlap on identity expression.

This analysis was repeated on the measure of perceived identity expression. The analysis revealed a significant main effect of identity overlap, *β* = 0.20, *p* = 0.03, 95% CI low = 0.01, high = 0.21, and a significant Overlap × Quantity interaction, *β* = -0.42, *p* < 0.001, 95% CI low = -0.08, high = -0.03. The main effect of identity quantity was significant at both low and high levels of identity overlap (**Figure 3**). At low identity overlap, identity quantity was positively associated with perceived identity expression, *β* = 0.28, *p* = 0.01, 95% CI low = 0.01, high = 0.08, whereas at high identity overlap, this relationship was negative, *β* = -0.47, *p* = 0.001, 95% CI low = -0.11, high = -0.02. Thus, having many different and distinct ways of identifying oneself seemed to facilitate the individual’s perceived ability to freely and clearly express their identities. Identifying with many overlapping social categories, however, appeared to inhibit perceived identity expression. The particular nature of the interaction should also be noted, though. Specifically, it would appear that people with few, but highly overlapping identities felt freer to express themselves than people with few, but non-overlapping identities, *t*(30) = -2.89, *p* = 0.01. This effect appeared to reverse as number of identities increased such that people with many distinct identities felt freer to express these identities than people with many overlapping ones. Although this trend is apparent the effect was not statistically significant, *t*(39) = 1.45, *p* = 0.15.

##### Social support

**FIGURE 4:**
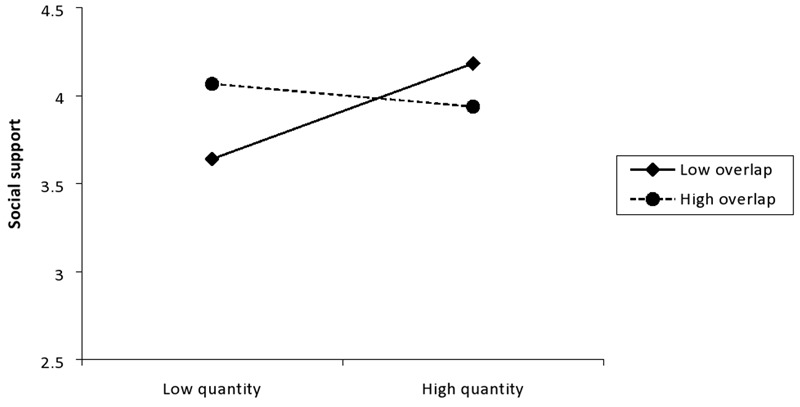
Interaction between identity quantity and overlap on perceived social support.

The analysis performed on the measure of social support, also generated effects comparable to those found for well-being. Again, although there were no significant main effects of either identity overlap or identity quantity, the Overlap × Quantity interaction was again significant, *β* = -0.21, *p* = 0.03, 95% CI low = -0.06, high = -0.01. Further analysis revealed that identity quantity was positively associated with social support at low, *β* = 0.30, *p* = 0.01, 95% CI low = 0.00, high = 0.06, but not high, *β* = -0.08, *p* = 0.59, 95% CI low = -0.04, high = 0.00, levels of identity overlap (see **Figure 4**).

We also assessed the extent to which the identity value variables (perceived by the self and others) moderated the relationship effects of identity quantity, identity overlap, and their interaction on well-being. These analyses revealed no significant effects, all *p*s > 0.50.

#### Mediation Analysis

Given the fact that our theoretical framework placed well-being as the ultimate outcome variable, we considered whether the effects of identity complexity on the DV were mediated by perceived identity expression and social support. To test the mediating role of perceived identity expression, this was included in the regression equation predicting well-being along with identity overlap, identity quantity, and the Overlap × Quantity interaction. In this analysis, the previously significant Overlap × Quantity interaction became non-significant, *β* = -0.08, *p* = 0.44. This was replaced by a significant main effect for perceived identity expression on well-being, *β* = 0.34, *p* < 0.001, suggesting that perceived identity expression mediated the effect of the Overlap × Quantity interaction on well-being.

**FIGURE 5:**
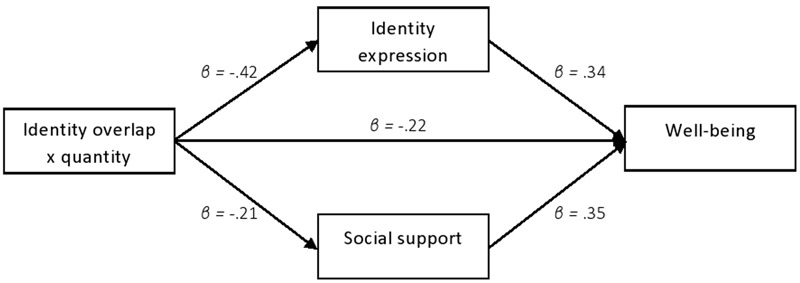
Relationship between identity overlap by identity quantity interaction and well-being as mediated by social support and identity expression.

The same analysis was repeated with social support included as a possible mediator. Similarly, the previously significant interaction became non-significant, *β* = -0.15, *p* = 0.10. Again, this interaction was replaced by a significant effect of the mediator (social support) alone, *β* = 0.35, *p* < 0.001, suggesting that social support also mediated some of the effects of the Overlap × Quantity interaction on well-being. This pattern of dual mediation via perceived identity expression and social support was confirmed in a bootstrapping analysis testing the significance of the indirect paths (Preacher and Hayes, 2008). Path analyses of the relationship are presented in **Table 3**, and the indirect effects of Overlap × Quantity on well-being via perceived identity expression and social support are shown in **Table 4**. Both indirect effects are significant at *p* < 0.05 (95% CI) (see **Table 4**). These relationships are depicted graphically in **Figure 5**.

**Table 3:** Impact of Quantity × Overlap on mediator variables and of mediator variables on well-being.

Path	Mediator	Coeff.	*SE*	*t*	*p*
IV to Mediators	Social support	-0.03	0.01	-2.31	0.02
	Identity expression	-0.05	0.01	-4.26	0.00
Mediators to DV	Social support	0.28	0.09	2.98	0.00
	Identity expression	0.20	0.09	2.20	0.03

**Table 4:** Indirect effects of Identity Quantity on Well-being via mediators and at different levels of Identity Overlap.

	Identity		Boot	Boot CI	Boot CI
Mediator	Overlap level	Effect	SE	(95%) low	(95%) high
Identity expression	Low	0.03^∗^	0.01	0.01	0.06
	High	-0.04^∗^	0.02	-0.08	-0.01
Social support	Low	0.03^∗^	0.01	0.00	0.06
	High	-0.01	0.01	-0.04	0.01

*5000 bootstrap samples, ^∗^significant at *p* < 0.05.*

### Discussion

The aim of Study 1 was to examine the relationship between multiple group memberships and well-being. In line with a growing body of research (Haslam et al., 2008; Binning et al., 2009; Jones and Jetten, 2011; Jetten et al., 2012), our results demonstrated that multiple group memberships contribute to well-being. But, importantly, this contribution was dependent on more than the sheer number of group memberships the individual has access to. Consistent with theorizing about SIC (Roccas and Brewer, 2002), the effect of multiple group memberships was contingent on perceived identity overlap. Specifically, it was multiple non-overlapping (i.e., distinctive) group memberships that contributed most positively to individual well-being.

Moreover, this effect was mediated by the individual’s perceived access to social support and the reported ease of self-expression. Consistent with the ideas presented in the introduction, it seems likely that belonging to social categories that are well defined and distinctive facilitates both the individual’s ability to articulate to their social world who they are, as well as their access to the social support that similar others might be able to provide. On the other hand, if social categories are highly overlapping, and thus consist of largely the same groups and people, both the ability to express the self clearly, and the ability to access multiple distinct sources of social support might be compromised. Beyond this, the psychological benefits of clear perceived identity expression can be explained in terms of self-verification processes. Past research suggests that people generally desire to be seen by others as they see themselves (i.e., self-verification), and that self-verification is psychologically a positive experience (Swann et al., 1989). Effective perceived identity expression is likely to be instrumental in attaining self-verification, and the process of enacting the self successfully to multiple others could help explain the relationship between perceived identity expression and well-being. The fact that perceived identity expression is facilitated by belonging to multiple distinctive groups, and undermined by membership in multiple overlapping groups, is an interesting extension of past work on these ideas. Specifically, these findings highlight the fact that multiple identities are not necessarily beneficial and in some instances maladaptive (i.e., when identities are non-distinct, possessing fewer identities appears to be better in terms of self-expression and through this well-being).

## Study 2

Study 1 highlights the importance of the *distinctiveness* of the groups to which the individual belongs for unlocking well-being benefits of multiple group memberships. Given our overarching aim to map out the factors that underpin the relationship between multiple group membership and well-being, however, we designed a second study looking at other potential mediators and moderators. In the introduction we highlighted additional features of multiple group membership that might also frame the relationship between these and well-being. In particular, we suggested how concerns around stigma can complicate the individual’s ability to enact group memberships and reap the associated benefits, and how stigma attached to one group membership might create incompatibilities with others. Thus, we proposed that belonging to multiple groups might not be so beneficial for well-being when the component groups are perceived to be devalued in the eyes of others (i.e., stigmatized: Schmitt et al., 2014; see also Brook et al., 2008).

Although we did ask about perceived social value in Study 1, it should be noted that most reported identities were perceived as valuable, both to the self and to others. In addition, the format of the questionnaire in Study 1, with its focus on uniqueness via questions about identity overlap, is likely to have prioritized concerns around the distinctiveness of the identities that comprise the self-concept more than questions of social stigma. In light of this, the aim of Study 2 was to shift the focus from questions about identity distinctiveness to the social *meaning* of identity in terms of social stigma, and to explore the consequences of this for relationships between multiple group memberships and well-being.

Given the above rationale, we again examined the relationship between multiple identities and well-being in Study 2. Here, however, our questions focused on the broader context of group memberships, rather than just their distinctiveness. Specifically, instead of asking people to compare their various group memberships in terms of overlap (which does not itself communicate social value), in this study we focused our questions on the visibility, value, and (in)compatibility between identities. Our reasoning was that assessing these features of identities, alone and in combination, should call to mind a different set of concerns than questions about overlap/distinctiveness. Specifically, questions about compatibility highlight the normative and socially determined aspects of multiple groups within the wider social environment (Brook et al., 2008). Similar social evaluative concerns are also activated by questions about the visibility of one’s identities to others, questions that were also not asked in Study 1. Indeed, past research suggests that belonging to visibly stigmatized groups (e.g., physically disabled, ethnic minority) as opposed to concealable ones (e.g., homosexuality) can have different consequences for individual well-being (Quinn, 2006; Quinn and Chaudoir, 2009; Quinn and Earnshaw, 2013). Thus, in this study, we re-framed our identity questionnaire in ways that allowed us to investigate additional features of identity that might play a role in determining links between multiple group memberships and well-being. Namely, the value of one’s identities in the eyes of others (i.e., visible vs. invisible identity value) and the degree to which those identities are compatible or not.

Consistent with past research, we expected that the well-being consequences of (multiple) group membership would be dependent on (1) the social value of these groups (i.e., whether some of these were stigmatized), (2) the degree to which these group memberships were obvious to others (visible vs. invisible), and (3) whether the group memberships and their associated identities were compatible. Specifically, we expected that multiple group membership would be positively related to well-being only when the component identities were visibly socially valued rather than visibly devalued. Membership in multiple visibly stigmatized groups was thus expected to undermine individual well-being. We further reasoned that belonging to multiple, stigmatized groups should be associated with a sense of incompatibility within one’s matrix of group memberships. This sense of incompatibility should, in turn, relate negatively to well-being. In addition, and similar to Study 1, we expected that possessing visibly stigmatized identities would strongly determine interactions with others and therefore make it difficult to express the self fully (i.e., in terms of the other identities that one might possess) and to access social support from others. Feeling unable to express one’s identities, and having lower perceived social support, were thus expected to mediate well-being. Finally, we also reasoned that one way in which identity stigma may negatively impact on the relationship between multiple group membership and well-being is through social exclusion. Specifically, being allowed access to the groups to which one belongs may become difficult if one or more of those groups is visibly stigmatized (Ellemers and Barreto, 2006). In order to test these ideas, we included a two-item measure of social inclusion. Thus, in this study, we test three possible mediators of well-being effects: Self-expression, social support, and social inclusion.

### Method

#### Participants

The survey was conducted online and advertised in an identical fashion to Study 1. A sample of 144 adults was recruited. Of these, 40 cases were missing data and were therefore not included. The final sample of 104 participants included 17 males and 86 females. The majority of these (55.6%, *n* = 58) were between 20 and 30 years old. A total of 20 different nationalities were included in the sample, with the majority 67.3% (*n* = 70) being from Australia (40.4%, *n* = 42) and the UK (26.9%, *n* = 28). The most common occupation was university student (63.5%, *n* = 66) followed by ‘other’ (13.5%, *n* = 14) and academic (11.5%, *n* = 12). The sample included 11 different ethnicities, but the vast majority of participants identified as White (76%, *n* = 70).

#### Survey and Measures

Similar to Study 1, participants were instructed to first list as many of their social identities that they could think of. They then chose the four most important identities before responding to a series of items focusing on various aspects of social identity, this time in terms of compatibility, visibility, and value. Participants then completed measures of psychological well-being.^2^

##### Identity measures

Identity quantity was measured in an identical fashion to Study 1. Identity visibility was measured for the top four identities with a single item, created for the study (‘To what extent do you feel that your membership with the category [X] is generally obvious to others?’ 1 = Not at all, 5 = Very much so). Similarly, the measure of identity value was gauged with a single item, ‘To what extent do you think your membership with [X] is considered positively or negatively by others in the community/society in which you live?’ The item was measured using a 5-point Likert scale with 1 = Generally negatively, 5 = Generally positively). We measured identity centrality using the same two-item measure as in Study 1 (α = 0.84).

In Study 1, participants were asked to compare important groups in terms of their overlap. In this study, those comparisons between important groups were instead based on perceptions of the compatibility between groups, something we argue is likely to have shifted the way identities were framed for the questions that followed, away from a focus on individual uniqueness (Study 1) and toward one of the social and normative meaning of identities. Specifically, the degree of perceived compatibility between each possible pairing of participants’ four most important identities (i.e., ‘Thinking about [group X] and [group X], how easy or difficult is it to belong to these two groups/social categories at the same time?’) was measured on 5-point Likert scales (1 = very difficult, 5 = very easy). The average compatibility score for all identity pairings was then calculated to obtain an overall measure of identity compatibility.

After rating each of their chosen identities on these dimensions, a number of more general questions about the self and identity were asked that did not refer to the specific groups. The Study 1 measures of perceived identity expression (α = 0.79) and perceived access to social support (α = 0.82) were used. Finally, we also included a two-item measure of perceived social inclusion that was developed for the study (scale reliability α = 0.93) (‘Generally, I feel included by my peers in the community,’ ‘Generally, I feel accepted by my peers in the community’). Psychological well-being was assessed using the same GWBI (Hopton et al., 1995) as in Study 1.

### Results

#### Descriptive Findings

On average, respondents listed a total of just over seven (*M* = 7.36, *SD* = 3.38) social identities that they believed defined them in some way. Similar to Study 1, participants reported a wide variety of groups, including gender, religion, profession, ethnicity, etc. The perceived compatibility of participants’ identities was generally high (*M* = 4.10, *SD* = 0.82), with the most compatible and incompatible identities exemplified by match-ups including *female* and *mother*, or *working class* and *Ph.D.-student*, respectively. Similarly, the other identity and outcome variables yielded average scores significantly higher than the scale midpoint of 3.00, except for perceived identity expression [*t* = -0.38 (103), *p* = 0.70; see **Table 5**]. Examples of highly visible identities, included those based on gender and ethnicity, while invisible identities related to, for instance, profession or politics. Minority ethnicity was also attributed low value, whereas *university graduate*, for example, was considered high value. In general, participants listed socially valued, highly visible, and compatible identities.

**Table 5:** Correlations between identity and well-being measures.

Variable	Mean	*SD*	1	2	3	4	5	6	7	8
(1) Identity quantity	7.36	3.38	–	0.07	0.09	0.23^∗^	-0.04	0.26^∗∗^	0.26^∗∗^	-0.01
(2) Identity compatibility	4.10^†^	0.82		–	0.19	0.12	-0.00	0.13	0.37^∗∗^	0.11
(3) Identity visibility	3.38^†^	0.82			–	0.76^∗∗^	-0.18	0.01	0.16	0.12
(4) Identity value	4.10^†^	0.64				–	0.02	0.14	0.28^∗∗^	0.07
(5) Identity expression	2.97	0.90					–	0.23^∗^	0.23^∗^	0.15
(6) Social support	4.17^†^	0.77						–	0.62^∗∗^	0.27^∗∗^
(7) Social inclusion	3.88^†^	1.01							–	0.29^∗∗^
(8) Well-being	3.48^†^	0.67								–

*^∗^*p* < 0.05, ^∗∗^*p* < 0.01. ^†^Mean departs from scale midpoint (3) significantly at *p <* 0.001.*

Next, mean correlations for the main identity and outcome variables were calculated (see **Table 5**). Significant positive correlations were evident between identity quantity and identity value (*β* = 0.23, *p* < 0.05), social support (*β* = 0.26, *p* < 0.05), and social inclusion (*β* = 0.26, *p* < 0.05). Similarly, identity compatibility (*β* = 0.37, *p* < 0.05) and identity value (*β* = 0.28, *p* < 0.05) were both positively associated with social inclusion. Perceived identity expression also correlated positively with both social support (*β* = 0.23, *p* < 0.05), and social inclusion (*β* = 0.23, *p* < 0.05). Interestingly, identity visibility and value correlated positively (*β* = 0.76, *p* < 0.01). While this association may at face value indicate that these constructs are in some way dependent, we argue that the high correlation is due to the notion that high-value identities are more likely to be rendered visible by the individual. That is, Christians may signal their religious identity openly (e.g., attending church service, wearing a cross, etc.) in a majority Christian society, but not in one critical of that particular faith. Overall, the participants who listed greater numbers of identities generally perceived their identities to be of high value, felt socially included, and had greater access to social support. Further, those who described their identities as highly compatible and of high value felt more socially included (see **Table 5**). However, and as in Study 1, identity quantity *per se* was not correlated with well-being. Similar to Study 1, we also examined the association between the identity and well-being variables at the level of each individual identity that participants listed (i.e., their four most important identities). These correlations largely mirrored those described above, with all but the last level of identity compatibility and first level of identity value correlating positively with social inclusion. There was also an inverse relationship between identity visibility and perceived identity expression at identity level 3 and 4 (see **Table 6**).

**Table 6:** Correlations between identity and well-being measures at the level of individual identities.

Variable	Level	Social support	Social inclusion	Perceived identity expression	Well-being
Identity compatibility	Identity 1, 2	0.11	0.28^∗∗^	-0.17	0.12
	Identity 1, 3	0.06	0.25^∗∗^	0.03	-0.07
	Identity 1, 4	0.10	0.27^∗∗^	-0.12	0.06
	Identity 2, 3	0.10	0.25^∗^	0.18	0.08
	Identity 2, 4	0.13	0.34^∗∗^	-0.02	-0.02
	Identity 3, 4	-0.01	0.17	0.02	0.11

Identity visibility	Identity 1	-0.05	0.09	-0.03	0.02
	Identity 2	-0.06	0.04	-0.10	0.07
	Identity 3	0.12	0.10	-0.22^∗^	0.05
	Identity 4	-0.00	0.17	-0.20^∗^	0.12

Identity visibility	Identity 1	0.08	0.12	0.01	-0.00
	Identity 2	0.21^∗^	0.28^∗∗^	-0.07	-0.02
	Identity 3	0.15	0.19^∗^	-0.00	-0.10
	Identity 4	0.28^∗^	0.42^∗∗^	-0.07	0.07

*^∗^Correlation is significant at the 0.05 level (two-tailed). ^∗∗^Correlation is significant at the 0.01 level (two-tailed).*

#### Regression Analyses

Regression analyses were undertaken to further determine the specific nature of the relationships among the variables. Well-being was used as the main DV, with social inclusion, social support, and perceived identity expression as secondary DVs. The IVs identity quantity, identity visibility, identity value, and identity compatibility and their interaction were of principal interest in accordance with the stated hypotheses. Regression analyses for all DVs were conducted in which the main effect terms were entered at the first step, followed by the interaction term at Step 2.

##### Well-being

There were no significant main effects of identity quantity (*β* = -0.03, *p* = 0.76, 95% CI low = -0.05, high = 0.03), identity visibility (*β* = 0.13, *p* = 0.19, 95% CI low = -0.08, high = 0.25), identity value (*β* = 0.05, *p* = 0.63, 95% CI low = -0.15, high = 0.27), or identity compatibility (*β* = 0.07, *p* = 0.43, 95% CI low = -0.10, high = 0.23) on well-being. Nor were there any interactions among these variables (all *p* > 0.05).

##### Social inclusion

**FIGURE 6:**
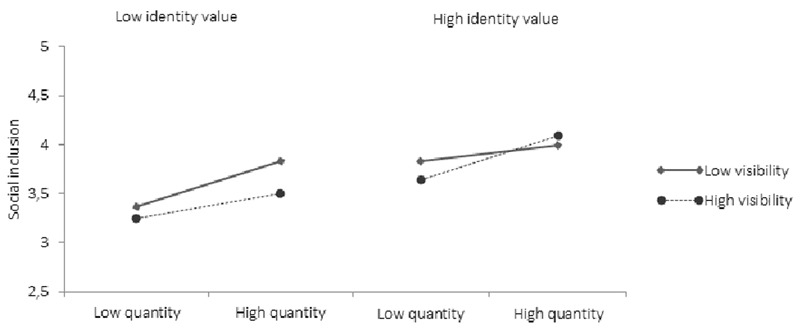
The association between identity quantity and perceived social inclusion at high and low visibility and value.

For social inclusion, main effects were evident for identity quantity (*β* = 0.19, *p* = 0.04, 95% CI low = 0.00, high = 0.10) and identity value (*β* = 0.23, *p* = 0.02, 95% CI low = 0.02, high = 0.60), as well as for identity compatibility (*β* = 0.31, *p* = 0.001, 95% CI low = 0.17, high = 0.61). Further, in line with our hypothesis that the relationship between multiple group membership and well-being was moderated by identity visibility and identity value, we tested a three-way interaction of identity quantity, identity value, and identity visibility. The interaction variable was significantly related to social inclusion (*β* = 0.15, *p* = 0.001, 95% CI low = 0.01, high = 0.29). To deconstruct the interaction, the correlation between identity quantity and social inclusion was examined at low and high identity value and visibility. When identity value was low (i.e., participants were reporting stigmatized identities), there was a significant positive relationship between identity quantity and social inclusion, but only when identity visibility was also low (*β* = 0.20, *p* = 0.00, 95% CI low = 0.01, high = 0.09) rather than high (*β* = -0.08, *p* = 0.19, 95% CI low = -0.07, high = 0.02). When identity value was high (i.e., non-stigmatized/positive), there were only weak (non-significant) positive relationships between identity quantity and social inclusion regardless of the level of identity visibility (Low: *β* = 0.05, *p* = 0.22, 95% CI low = -0.01, high = 0.04; High: *β* = 0.08, *p* = 0.07, 95% CI low = -0.01, high = 0.06; see **Table 7** and **Figure 6**). Thus, multiple group memberships contributed to a sense of social inclusion most for individuals with stigmatized but invisible identities.

**Table 7:** Impact of three-way interaction on mediators, and mediators on outcome variable (well-being).

Path	Mediator	Coeff.	*SE*	*t*	*p*
Interaction variable to mediators	Social support	0.14	0.04	3.57	0.00
	Social inclusion	0.15	0.05	3.06	0.00
	Identity expression	0.08	0.04	2.29	0.02
Mediators to outcome variable	Social support	0.26	0.09	2.79	0.01
	Social inclusion	0.22	0.07	3.04	0.00
	Identity expression	0.34	0.09	3.80	0.00

##### Social support

**FIGURE 7:**
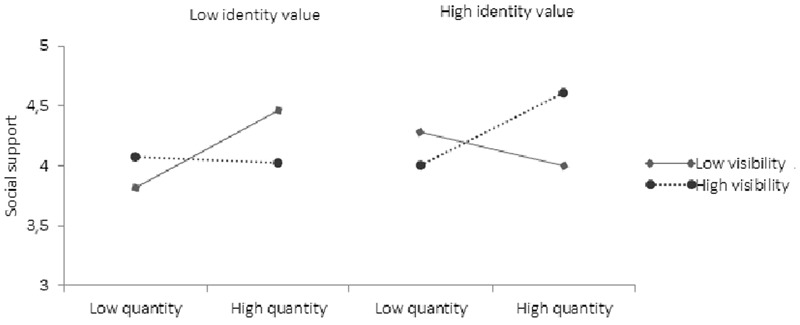
The association between identity quantity and perceived social support at high and low visibility and value.

The analysis on social support revealed main effects of identity quantity (*β* = 0.22, *p* = 0.03, 95% CI low = 0.01, high = 0.10), but not identity visibility (*β* = -0.01, *p* = 0.89, 95% CI low = -0.19, high = 0.16), identity value (*β* = 0.07, *p* = 0.50, 95% CI low = -0.19, high = 0.16), or identity compatibility (*β* = 0.09, *p* = 0.29, 95% CI low = -0.08, high = 0.20). However, identity quantity, visibility, and value again interacted to impact significantly on social support (*β* = 0.14, *p* < 0.001, 95% CI low = 0.00, high = 0.08). Deconstructing the interaction, we examined the relationship between identity quantity and social support at low and high identity value and in turn at low and high levels of identity visibility. This revealed several significant main effects. Replicating the previous pattern, when identity value was low (stigmatized identities), there was a significant positive relationship between identity quantity and social support when identity visibility was also low (*β* = 0.14, *p* = 0.01, 95% CI low = 0.01, high = 0.09), but not when identity visibility was high (*β* = 0.02, *p* = 0.55, 95% CI low = -0.05, high = 0.01). When identity value was instead high, there was a significant positive relationship between identity quantity and social support when identity visibility was also high (*β* = 0.12, *p* = 0.00, 95% CI low = 0.00, high = 0.08), but not when visibility was low (*β* = -0.06, *p* = 0.28, 95% CI low = -0.01, high = 0.03) (see **Figure 7**). The results thus indicated a positive correlation between identity quantity and social support for people with stigmatized, but invisible, identities, and for people with valued and visible identities.

##### Perceived Identity expression

**FIGURE 8:**
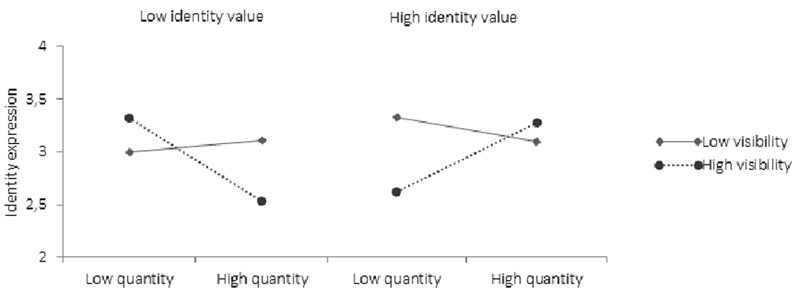
The association between identity quantity and ease of identity expression at high and low visibility and value.

The analysis on perceived identity expression, revealed only a significant three-way interaction between identity quantity, identity value, and identity visibility (*β* = 0.13, *p* < 0.01, 95% CI low = 0.01, high = 0.07). When identity value was low (i.e., stigmatized identities), identity quantity was negatively correlated with perceived identity expression when identity visibility was also high (*β* = -0.20, *p* = 0.00, 95% CI low = -0.06, high = -0.00), but not when visibility was low (*β* = -0.00, *p* = 0.20, 95% CI low = -0.03, high = 0.02) (see **Table 7** and **Figure 8**). Further, when identity value was high, identity quantity correlated positively with perceived identity expression when identity visibility was also high (*β* = 0.10, *p* = 0.03, 95% CI low = 0.00, high = 0.04), but not when visibility was low (*β* = 0.05, *p* = 0.21, 95% CI low = -0.01, high = 0.01). As such, multiple identities inhibited social expression when identities were stigmatized and visible. When identities were socially valued and visible, however, the quantity of identities facilitated expression.

##### Identity compatibility

**FIGURE 9:**
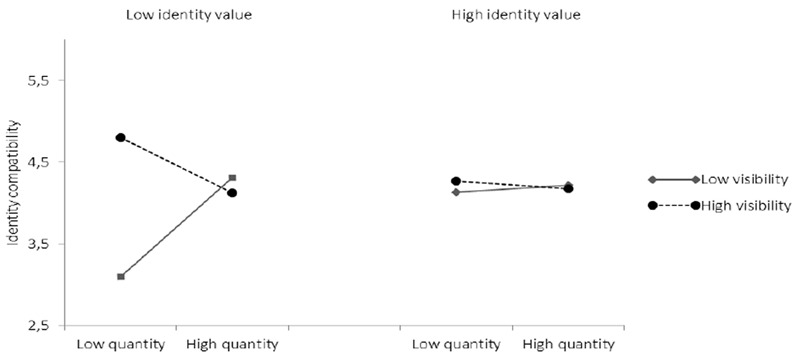
The association between identity quantity and perceived identity compatibility at high and low visibility and value.

Given the lacking contribution of identity compatibility as a moderator variable in the above analyses, we explored the possibility that perceived compatibility might be an outcome of the features of identities – that is, that possessing stigmatized identities might give rise to a sense of identity incompatibility, mediating the effects of stigma on well-being. Thus, we theorized that stigmatized identities may be more difficult to square with other identities in the self-concept, thus creating a sense of incompatibility. While there were no significant main effects of identity quantity, identity value, or identity visibility, the three-way interaction was again significant, *β* = 0.12, *p* = 0.00, 95% CI low = 0.00, high = 0.10. When identity value was low (i.e., stigmatized identities), identity quantity was positively related to identity compatibility when identity visibility was also low (*β* = 0.18, *p* = 0.00, 95% CI low = 0.00, high = 0.13). However, this relationship reversed in the context of low value, but high visibility identities (*β* = -0.10, *p* = 0.06, 95% CI low = -0.10, high = -0.00) (see **Table 7** and **Figure 9**). Thus, having relatively many identities facilitated a sense of identity compatibility when identities were stigmatized but invisible to others. The opposite was true, when stigmatized identities were visible, although this relationship was weaker and only marginally significant.

While the above analyses are interesting and in line with predictions, several somewhat surprising patterns should be noted. Specifically, it would appear that people who had relatively few identities were worse off in terms of social support, perceived identity expression, and compatibility when these identities were devalued and invisible than if they were devalued and visible (see **Figures 7–9**). The comparable effects on these variables appeared to reverse, however, as the number of identities increased, such that people with many identities had access to more social support and found it easier to self-express if those identities were devalued and invisible rather than devalued and visible. Speculatively, this suggests that certain forms of stigma can also be self-protective (e.g., Crocker and Major, 1989).

#### Mediation Analysis: Social Inclusion, Social Support, Perceived Identity Expression

**FIGURE 10:**
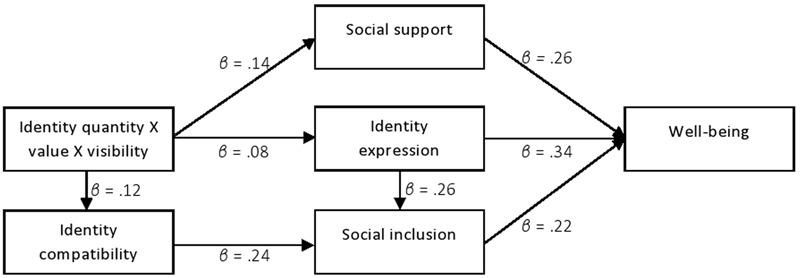
Relationship between the three-way interaction of identity quantity, identity visibility on well-being as mediated by social support, social inclusion, identity compatibility and identity expression.

Despite the absence of any association between the IVs and the key DV (well-being), we nonetheless explored the patterns on the expected mediator variables of social inclusion, social support, and perceived identity expression. Past research has advocated testing for mediation in the absence of direct effects by bootstrapping on the grounds that the causal steps approach is considerably low in power, and thus relatively unlikely to detect an effect. In addition, when research is guided by a theoretical model, it is preferable to test the degree to which that model – as originally conceived – fits the data. Although it may seem counter-intuitive to do this when there are no discernible direct effects on the dependent measure, the absence of these also does not preclude validity of the original model (Hayes, 2009, 2012; Hayes and Preacher, 2014). Because PROCESS does not allow multiple parallel mediators in models that involve interactive IVs, we tested each mediator separately. Thus, we report the results of five mediation models. As hypothesized, the results of three mediation models run using PROCESS (Hayes, 2012; Models 3 and 12), indicated indirect effects of the interaction variable on well-being via social support, social inclusion, and perceived identity expression, respectively. Further, in another two models, we found that perceived identity expression and identity compatibility, respectively mediated the relationship between the interaction variable and social inclusion. We explain this with the idea that belonging to potentially incompatible groups (e.g., gay and Muslim) will likely make it difficult for the individual to express the associated identities equally and freely, and obstruct his or her inclusion in either or both of these groups (Jaspal and Cinnirella, 2010). We have integrated each of our mediation models into a single figure for a complete representation (**Figure 10**), and presented the appropriate statistics in **Tables 7–10**.

These mediation effects were confirmed in bootstrap analyses testing the significance of the indirect paths. Path analyses and the indirect effect of identity quantity × identity value × identity visibility on well-being via perceived identity expression, social inclusion, and social support are presented in **Tables 7, 8**. Path analyses and the indirect effect of the interaction variable via perceived identity expression and identity compatibility on social inclusion are shown in **Tables 9, 10**, respectively. The indirect relationships for well-being are significant at *p* < 0.05 (social support, boot 95% CI low = 0.01, high = 0.01; social inclusion, boot 95% CI low = 0.00, high = 0.09; perceived identity expression, boot 95% CI low = 0.00, high = 0.06) as are those for social inclusion (perceived identity expression, boot 95% CI low = 0.0049, high = 0.0964; identity compatibility, boot 95% CI low = 0.0025, high = 0.0838).

**Table 8:** Indirect effects of identity quantity on well-being via mediators, and at different levels of social acceptance and identity visibility.

	Identity	Identity		Boot	Boot CI	Boot CI
Mediator	value	Visibility	Effect	*SE*	(95%) low	(95%) high
Social support	Low	Low	0.04^∗^	0.02	0.01	0.09
		High	-0.01	0.02	-0.06	0.01
	High	Low	0.01	0.01	-0.01	0.03
		High	0.03^∗^	0.02	0.00	0.09
Social inclusion	Low	Low	0.05^∗^	0.02	0.01	0.10
		High	-0.02	0.02	-0.07	0.02
	High	Low	0.01	0.01	-0.01	0.04
		High	0.01^∗^	0.01	0.00	0.05
Identity Expression	Low	Low	-0.00	0.01	-0.05	0.02
		High	-0.04^∗^	0.02	-0.09	-0.00
	High	Low	-0.00	0.01	-0.01	0.01
		High	0.03^∗^	0.01	0.01	0.06

*^∗^*p* < 0.05.*

**Table 9:** Impact of three-way interaction on mediators, and mediators on social inclusion.

Path	Mediator	Coeff.	*SE*	*t*	*p*
Interaction variable to mediators	Identity Compatibility	0.12	0.04	2.93	0.00
	Identity Expression	0.08	0.04	2.29	0.02
Mediators to outcome variable	Identity Compatibility	0.24	0.12	1.96	0.05
	Identity Expression	0.26	0.10	2.49	0.01

**Table 10:** Indirect effects of identity quantity on social inclusion via mediators, and at different levels of social acceptance and identity visibility.

	Identity	Identity		Boot	Boot CI	Boot CI
Mediator	value	Visibility	Effect	*SE*	(95%) low	(95%) high
Identity Expression	Low	Low	-0.01	0.03	-0.04	0.05
		High	-0.05^∗^	0.03	-0.12	-0.00
	High	Low	-0.00	0.01	-0.02	0.02
		High	0.02^∗^	0.02	0.00	0.06
Identity Compatibility	Low	Low	0.04^∗^	0.02	0.01	0.14
		High	-0.02^∗^	0.02	-0.08	-0.00
	High	Low	0.00	0.01	-0.02	0.04
		High	-0.00	0.01	-0.04	0.03

Finally, we regressed well-being onto all of the IVs and their interaction, with the mediator variables simultaneously included as predictors of well-being. As expected, we found a significant association between the outcome variable and social support (*β* = 0.27, *p* = 0.005) and social inclusion (*β* = 0.32, *p* = 0.003). Perceived identity expression was marginally significantly associated with well-being (*β* = 0.18, *p* = 0.08), while the relationship between identity compatibility and well-being was non-significant. Identity compatibility did, however, significantly mediate social inclusion (*β* = 0.31, *p* = 0.001), as did perceived identity expression (*β* = 0.26, *p* = 0.004).

### Discussion

As a preamble to our discussion of the results of Study 2, we feel the need to restate the exploratory nature of the present research. Specifically, we point out the obvious limitation of conducting three-way interactions with our relatively small sample size, and thus recommend caution in the interpretation of our results. In comparison to Study 1, which foregrounded questions about the overlap between, and therefore distinctiveness of, the individual’s configuration of group memberships, Study 2 focused respondents more on the socially prescribed features of group memberships (i.e., the degree to which it is hard to belong to two groups simultaneously). This, we argue, is likely to have brought any concerns around stigma to the fore when individuals contemplated multiple group memberships and their self-concept in relation to these. In line with the assumed focus on stigma in this study, the results revealed consistent interactions among the number of groups to which an individual belonged and the social value and visibility of those groups. Multiple group memberships appeared to be a resource especially for individuals who belonged to groups that were relatively devalued (i.e., stigmatized) but otherwise invisible to others. Under these conditions (i.e., invisible stigma), belonging to multiple groups enhanced the ability to express the self, increased perceived access to social support as well as the individual’s sense of social inclusion. In comparison, when group memberships were stigmatized and visible, the benefits of multiple group memberships for social inclusion and social support did not accrue, and multiple group membership undermined the perceived ability to express one’s identities. A parallel pattern was observed on perceived identity compatibility. Conversely, in the context of valued identities, the benefits of multiple group memberships seemed more straightforward, and if anything were enhanced by the visibility of those valued identities.

Although there were no direct effects of the IVs on our ultimate DV, well-being, analysis of the indirect paths revealed a similar pattern of mediation to Study 1. Again, the extent to which multiple group memberships contributed to perceived identity expression and social support indirectly determined their benefits for well-being. Elaborating this picture, identity compatibility and perceived identity expression mediated feelings of social inclusion, which in turn also mediated well-being effects.

While the results are broadly consistent with expectations, certain unexpected and counter-intuitive patterns should be noted. Specifically, for people with few and stigmatized group memberships, feelings of compatibility, self-expression, and social support were greater if the associated identities were visible rather than invisible (**Figures 7–9**). Given their unexpected nature, it would be unwise to over-interpret these patterns. Nonetheless, in terms of social support and perceived identity expression, this could speculatively be due to the social isolation arising as a consequence of belonging to few and stigmatized identities which are also invisible. Under these circumstances individuals might feel especially alone. The ability and willingness to reach out and connect with similar others for support, might be hindered when this involves exposing devalued and concealed identities to others, especially when one has little else to draw on for one’s self –concept (Barreto and Ellemers, 2003). By contrast, belonging to a devalued but visible group, represents a situation in which the individual has no control over the prominence of his or her stigmatized identity. In the context of a self-concept comprising only few group memberships, this may be a relatively good thing, as self-expression and accessing social support (perhaps more from similar others; Frable et al., 1998) is likely to be relatively clear-cut. Indeed, some past research suggests that stigma can sometimes be self-protective (Crocker and Major, 1989), and that one way in which individuals might deal with devaluation from others is through defining themselves more strongly in terms of that stigmatized identity (Schmitt et al., 2003). In this way, being defined, and defining one’s self, in terms of simple yet stigmatized identities might not always be psychologically costly. Similarly, with respect to identity compatibility, belonging to few groups that are highly visible and stigmatized leaves the individual with little other choice than to accept their obvious membership in these groups, and somehow combine the associated identities into a congruous self-concept. If these identities are invisible, however, the individual may not feel the same outside pressure or need to merge their identities into a cohesive self, leaving the incompatibility unresolved. Of course, the correlational and self-reported nature of our study also leaves open the possibility that those who are most vulnerable – for example, those with a very singular and stigmatized sense of self – might also withdraw from the identities involved, and from questions about them. This could inflate the apparent value of being visibly stigmatized and relatively isolated because it is only those who have come to terms with this situation who are also willing to report their experiences. Similarly, those identities that are visible yet stigmatized (e.g., ethnicity in our sample), differ on many dimensions to those identities that are stigmatized yet invisible (e.g., mental illness (specifically depression) in our sample). It is also plausible that some of these unexpected findings stem from the specifics of the identities people were contemplating. Our approach, which collapses across specific group memberships to consider broad features of relevance to stigma and identity, necessarily loses some of this important detail.

## General Discussion

The present research focused on the relationship between multiple group membership and well-being. Although past findings suggest a positive relationship between these constructs, the focus of our two studies was to explore in more detail the complexity of identities and how this might shape the benefits of these when they are combined. Specifically, in two studies, we explored how the benefits of multiple group membership might depend on the extent to which individual component identities are distinct (Study 1), and on how concerns arising from social stigma, and associated identity incompatibilities, may disable the benefits of multiple group memberships (Study 2).

Study 1 showed that the advantages of multiple group membership for well-being were moderated by SIC (Roccas and Brewer, 2002). Membership in multiple groups promoted individual well-being most when (the most central) individual group memberships were perceived to be non-overlapping and distinct (i.e., high SIC). The effect of multiple group membership in combination with SIC was mediated through perceived identity expression and social support. That is, individuals with multiple group memberships characterized by distinctiveness also felt more confident in how they could express their self to others and perceived more sources of social support, both of which contributed positively to well-being.

In Study 2, we aimed to activate a different interpretive framework for individuals as they contemplated their multiple group memberships and what these might mean for their self. Rather than highlighting questions about overlap and the distinctiveness of one’s identity combinations, the questions in our second study highlighted issues of social stigma, including the value and visibility of one’s identities to others, as well as the perceived conflict between one’s identity combinations. In this study, individuals benefited most from multiple group membership when their (most central) identities were stigmatized, but invisible, or valued and visible to others. Further, the results indicated that any well-being consequences of identities were indirect and mediated through perceived identity expression and social support (as in Study 1), as well as social inclusion and, to a lesser extent, identity compatibility.

Integrating the results from Studies 1 and 2, these findings add to previous discussion and general theory of the benefits of multiple group membership for individual well-being (Roccas and Brewer, 2002; Iyer et al., 2009; Jones and Jetten, 2011). As outlined in the Introduction, past research has tended to focus on the number of multiple identities held in the self-concept as a central determinant of well-being (Jetten et al., 2010; Jones and Jetten, 2011). While other studies have suggested the significance of the specific features and characteristics of social identities in terms of their well-being benefits, these have seldom been incorporated in research on the benefits of *multiple* group memberships. The mechanisms by which the previously observed positive relationship between multiple group membership and well-being is facilitated, or undermined, are thus somewhat unclear. Our research, sheds some light on the specific nature of this association. While Study 1 demonstrates the general value of multiple *distinctive* identities, Study 2 highlights how the benefits of multiple identities are contingent on socially prescribed identity characteristics such as stigma and incompatibility. In so doing, the results generated by these two studies together identify a set of factors that, in different situations and capacities, may both facilitate and impede the previously demonstrated benefits of belonging to multiple groups.

Considering the patterns across studies – patterns that were simultaneously convergent (with respect to the underlying processes) and divergent (with respect to which factors moderated the impact of multiple group memberships) – it could be suggested that each study foregrounded different frameworks that guided how individuals interpreted the meaning of their group memberships. Study 1 focuses purely on the *descriptive* facets of group memberships and their boundaries – that is, who is and who is not a member of various groups? Within this framework, it would appear that the more and the more distinct the component identities, the better off the individual is in terms of well-being, while the more and the more overlapping the identities, the worse off the individual is. Study 2, on the other hand, emphasizes the *social meaning* of group memberships (rather than their boundaries and membership overlap) by looking at how identities are viewed by others and whether it is simple or complicated to belong to multiple groups at once. Once the social meaning of these group memberships is accounted for in this way, the nature of the relationship changes and the benefits of group membership becomes more defined by the social context in which the individual exists and the social perception of the given group memberships. That is, in this framework the well-being consequences of multiple group membership is more about what the current social environment permits based on the socially determined value of the specific groups to which the individual belongs. Thus, beyond that of the individual’s own interpretation and acknowledgment of the boundaries of his or her group memberships (i.e., distinctiveness vs. overlap), the present broader social environment also features as both a potential obstacle, inhibitor (in terms of compatibility), and facilitator of the benefits of group membership.

### Limitations and Future Directions

While these two studies have highlighted different ways in which multiple group memberships might affect well-being, our results are by no means exhaustive. Importantly, the findings we have offered are novel, and therefore tentative, especially given the relatively small samples we have drawn on to test our predictions. While we believe that there are sensible theoretical foundations for the relationships we describe, the estimates of these effects from the current data are likely to be imprecise and potentially unstable. As such, further empirical investigation is needed. Our more modest hope is that by discussing the different ways in which multiple groups might contribute to individual well-being, and providing demonstrations of divergent possibilities, future researchers will take up these ideas and elaborate on the critical questions of when multiple groups are a benefit, and when they are a burden, to the individual.

Our research designs also preclude asking more complicated questions about the place of multiple group memberships within individual selves. For example, our studies did not allow for any extensive insight into the internal negotiation of multiple identities in participants. In the context of many vs. few identities, it would be interesting to look at how individuals reason and negotiate concealable vs. obvious stigmatized identities or overlapping vs. distinct identities, and whether this may have an impact on the well-being effects of multiple group memberships. This could allow for a greater understanding of our somewhat counter-intuitive findings where people with few and highly overlapping or visibly stigmatized identities somehow were better off than people with few and distinct or valued identities.

Further, the method used to measure identity centrality, value, and visibility was based on averaging the individual scores for the four identities that participants were asked about. Acknowledging the fact that people might belong to groups that differ greatly in terms of these characteristics, this method might be somewhat problematic. For example, the effects of belonging to a heavily stigmatized category might not register proportionately if the individual rating of the associated identity is counterweighed by three positive or even neutral identities. Especially in terms of identity visibility, averaging presents a special problem by not accounting for exactly which identities are visible or invisible. As our data indicates, this matters a great deal when it comes to identity value. In other words, using averages to estimate overall identity value, visibility, and importance, might muddle the true effects of these variables. However, we also reasoned that interpreting the data at an identity level would be similarly complicated as we effectively would be comparing across participants, and thus across varied identities. Considering the fact that we found significant correlations in spite of these limitations, one could speculate that our results would only strengthen if these methodological issues were accounted for. This, of course, remains to be empirically tested.

We also acknowledge two limitations related to the measure of identity overlap. First, as outlined in the methods section, in line with Roccas and Brewer (2002), we asked participants to gauge the overlap between each pair of chosen identities. However, in order to keep the survey as short as possible and minimize the risk of participant fatigue, we chose to instruct participants only to estimate the overlap between, for example, Groups 1 and 2 and not vice versa. As such, this might only tell part of the story if the degree of overlap is dependent on which group is compared to the other. That is, most (religious) Italians might be Catholic, but most Catholics are not Italian. Again, exploring the details of individual identities and their combinations is something for future research. However, as a broad starting point, these studies suggest value in pursuing these questions further.

Another limitation relates to the correlational method employed that limits our ability to make statements about the causal directions of the observed relationships. Thus, while we have defined particular variables as antecedents, mediators, and outcomes on a theoretical basis, there is no empirical evidence of causality in the relationships found. Future study should therefore attempt experimental longitudinal research designs to tease out causal connections between multiple group membership and well-being variables. This could be done, for example, by replicating and elaborating on the experimental design of Jones and Jetten (2011), which activated different numbers of groups before assessing physical resilience. Adapting this paradigm, future studies could manipulate the number and meaning of salient identities to explore their combined effects on indicators of well-being.

Finally, it might be productive for future research to move beyond broad measures of well-being and to delve more deeply into the processes behind those feelings. In that regard, the use of more objective outcome variables, such as for example physiological measures of well-being (e.g., indexed by stress responses and resilience, see Blascovich et al., 2011), might be useful to supplement findings based on self-report scales. For example, past research has indexed physiological arousal in terms of the extent to which the participant perceives something as a threat or a challenge, and linked such responses to mental well-being (e.g., loneliness, Cacioppo et al., 2002; Hawkley et al., 2003) and self-esteem (Seery et al., 2004). Indeed, there is some evidence that these kinds of physiological reactions are causally influenced by multiple group memberships (Jones and Jetten, 2011).

Other questions arising from our research concern the connection between the three central identity features discussed above. For example, how do SIC and identity compatibility relate to one another? The somewhat paradoxical notion that multiple overlapping identities (generally a bad thing) are likely to also be compatible (generally a good thing) whereas many disparate identities are more likely to give rise to incompatibility, seems theoretically reasonable. Exactly how the descriptive features of groups relates to the subjective experience of group membership is, however, likely to be complex – especially when considering counter examples of highly overlapping, but nonetheless incompatible identities, including for example, women in the workforce. That is, there are many women who have careers, and these two categories (women and workers) thus overlap considerably. However, they are also perceived as somewhat incompatible (Burgess and Borgida, 1999; Schneider and Northcraft, 1999; Fiske et al., 2002). In light of these apparent contradictions, it seems pertinent to look at exactly how different, but potentially related, identity structures interact and affect individual well-being, and specifically to attend to the difference between what is (i.e., who factually belongs to this group and that group) and what *should* be (i.e., whether belonging to these two groups is socially permitted and subjectively experienced as such).

## Conclusion

The results reported here demonstrate that the relationship between multiple group memberships and well-being is neither straightforward nor linear. Instead, we have presented evidence showing that the quantity of identities available to a person does predict well-being, but that this link is dependent on the specific features of identities being combined. When the individuals’ most important group memberships signal distinctiveness, membership in multiple groups seems to contribute positively to well-being. When the individual’s most important group memberships are overlaid with concerns around stigma, membership in multiple groups might instead detract from sources of well-being. Interestingly, and despite these divergent patterns, the pathways between multiple group memberships and well-being were consistently found to be mediated through individual perceived identity expression and access to social support, as well as related processes like social inclusion. In some ways, these processes underpinning the relationship between multiple group membership and well-being might be the most important findings of this study. First, they link the individual perception of the categories to which one belongs (i.e., identity distinctiveness) with the outward enactment of the associated identities (perceived identity expression and social support), and consequent benefits (well-being). Second, they highlight the importance of the socially anchored meaning of group memberships (stigmatization and its visibility) to the way in which we are able to present ourselves socially (expression), engage with others productively (social support), and integrate multiple identities into a coherent and supportive self-concept (identity compatibility). Identifying these processes opens up the possibility of a deeper understanding of how, and under which conditions, multiple group memberships support and protect vs. undermine and fragment the self, and of the costs and benefits this may have for personal well-being.

## Ethics Statement

This study was carried out in accordance with the recommendations of Economic and Social Research Council and the Psychology Ethics Committee of the University of Exeter with written informed consent from all subjects. All subjects gave written informed consent in accordance with the Declaration of Helsinki. The protocol was approved by the Psychology Ethics Committee of the University of Exeter.

## Data Availability

The research materials supporting this publication can be accessed by contacting Dr. Anders Larrabee Sønderlund at sonderlund@gmail.com.

## Author Contributions

AS, TM, and MR all made substantial contributions to the conception, design, analysis, and interpretation of the work. The work was initially drafted by AS and then critically revised for important intellectual content by TM and MR. AS, TM, and MR all approved the version of the manuscript to be published, and all three authors agree to be accountable for all aspects of the work in ensuring that questions related to the accuracy or integrity of any part of the work are appropriately investigated and resolved.

## Conflict of Interest Statement

The authors declare that the research was conducted in the absence of any commercial or financial relationships that could be construed as a potential conflict of interest. The reviewer TK declared a shared affiliation, though no other collaboration, with one the authors MR to the handling Editor, who ensured that the process nevertheless met the standards of a fair and objective review.

## Footnotes

1 All measures and manipulations in these studies are reported in this paper.

2 All measures and manipulations in these studies are reported in this paper.
